# Impact of the treatment of pancreatic exocrine insufficiency on survival of patients with unresectable pancreatic cancer: a retrospective analysis

**DOI:** 10.1186/s12885-018-4439-x

**Published:** 2018-05-05

**Authors:** Juan Enrique Domínguez-Muñoz, Laura Nieto-Garcia, Javier López-Díaz, Jose Lariño-Noia, Ihab Abdulkader, Julio Iglesias-Garcia

**Affiliations:** 1 Department of Gastroenterology and Hepatology, University Hospital of Santiago de Compostela, C/ Choupana s/n, 15706 Santiago de Compostela, Spain; 2 Health Research Institute (IDIS), University Hospital of Santiago de Compostela, C/ Choupana s/n, 15706 Santiago de Compostela, Spain; 3 Department of Pathology, University Hospital of Santiago de Compostela, C/ Choupana s/n, 15706 Santiago de Compostela, Spain

**Keywords:** Pancreatic carcinoma, Pancreatic exocrine insufficiency, Malnutrition, Survival, Unresectable pancreatic cancer, Pancreatic enzyme replacement therapy

## Abstract

**Background:**

Malnutrition and weight loss are commonly observed in patients with pancreatic cancer and contribute to poor survival. Pancreatic exocrine insufficiency (PEI), which can be caused by ductal obstruction by a tumor, causes maldigestion and malabsorption of nutrients, thus contributing to malnutrition in these patients. In this study, we evaluated the effects of pancreatic enzyme replacement therapy (PERT) on survival in patients with unresectable pancreatic cancer.

**Methods:**

A retrospective analysis was conducted on a database of patients with unresectable, pathologically confirmed pancreatic cancer. All patients were evaluated for palliative chemotherapy and received the optimal palliative care. Patients were divided into two groups: Group 1 received standard therapy; Group 2 underwent additional evaluation of the pancreatic function and therapy with PERT, if needed. Survival (median and 95% confidence interval [CI]) was analyzed using Kaplan–Meier and Cox regression; groups were compared using the log-rank test.

**Results:**

Overall, 160 patients with unresectable pancreatic cancer were included in the analysis (mean age: 70.5 years [range 28–100]; gender: 57.5% male; tumor stage: 78.7% Stage IV). Eighty-six patients (53.75%) were in Group 1 and 74 (46.25%) were in Group 2. Age, gender, tumor size, location and stage, weight loss, and serum CA 19–9 were similar between groups. Ninety-three (58.1%) patients received palliative chemotherapy; 46.5% in Group 1 and 71.6% in Group 2 (*P* < 0.001). Forty-nine (66.2%) patients in Group 2 and none in Group 1 received PERT. Survival in Group 2 (189 days, 95% CI 167.0–211.0 days) was significantly longer than in Group 1 (95.0 days, 95% CI 75.4–114.6 days) (HR 2.117, 95% CI 1.493–3.002; *P* < 0.001). Chemotherapy and PERT were significantly and independently associated with longer survival in a model controlled by age and tumor stage. In patients with significant weight loss at diagnosis (> 10% bodyweight within 6 months), PERT was associated with longer survival (HR 2.52, 95% CI 1.55–4.11; *P* < 0.001).

**Conclusions:**

In patients with unresectable pancreatic cancer, PERT in patients with PEI was associated with longer survival compared with those not receiving PERT, especially in those experiencing significant weight loss. This finding should guide future prospective clinical trials of similar interventions.

## Background

Pancreatic cancer is the fourth-leading cause of cancer-related deaths in the United States [1] and is expected to become the second most common cancer by 2030 [2]. Patients presenting with pancreatic cancer typically have a poor prognosis, because of the advanced stage of the disease at the time of diagnosis. Pancreatic cancer is associated with a very high mortality rate [3]; an overall 5-year survival rate of 7% after diagnosis was reported over the 2004–2010 period [1]. This survival rate was further reduced to 2% for patients with advanced disease [1].

In patients with early-stage, resectable disease (15–20% of patients at time of diagnosis [4]), surgical resection of the tumor can be performed and, although associated with a low success rate, it represents the only potentially curative intervention. Median survival for resected patients is in the region of 14–20 months [5]. Five-year survival rates of up to 25% have been reported following pancreatectomy for ductal adenocarcinoma of the pancreas [5–8]; however, these values have been questioned and actual rates are believed to be much lower [9–12].

Approximately 80% of patients with pancreatic cancer present with either locally advanced or metastatic disease at time of diagnosis and are not suitable for surgery. Median survival for these patients is shorter (8–12 months for unresectable disease [13] and less than 6 months for metastatic disease [3, 10]).

Despite extensive research efforts over the last two decades, palliative treatment (e.g., chemotherapy and radiotherapy) has not significantly improved survival [14], quality of life and tumor progression for patients with unresectable pancreatic cancer. In recent years, however, the clinical relevance of malnutrition caused by pancreatic exocrine insufficiency (PEI) both in resected patients and in patients with advanced, unresectable pancreatic cancer, has been investigated to improve supportive care [15–17].

Malnutrition and significant weight loss are frequently observed in patients with pancreatic cancer [18] and influence survival, resection rate, tumor progression, and quality of life [5, 19]. Improvement of the nutritional status by parenteral nutrition enhances quality of life, and allows for tumor therapy to be administrated without interruption [20].

While malnutrition and weight loss may be partly attributed to anorexia, diarrhea, early satiety [21], chemotherapy, and/or tumor progression [20], PEI may also be a determinant, either due to postoperative changes in the digestion process after tumor resection, or to the tumor itself obstructing the pancreatic duct [22–24]. PEI causes digestion to be impaired, which commonly manifests as diarrhea and steatorrhea [25]. Reduced digestion results in malabsorption of nutrients, leading to malnourishment [18, 25]. A markedly reduced exocrine pancreatic secretion, as measured by low fecal elastase-1 (FE-1) levels, has been reported as an independent factor associated with poor prognosis in patients with advanced pancreatic cancer [26]. In this patient population, partial pancreatic resection for pancreatic malignancy leads to sustained PEI and reduced quality of life [27].

Oral pancreatic enzyme replacement therapy (PERT) is the standard treatment for PEI [21, 23]. Patients with advanced pancreatic cancer who received PERT have shown significant weight gain after PERT [16]. In addition, PERT was associated with significant improvement in fat and protein digestion in patients with a partial/total pancreatic resection [28]. Of note, a recent study reported low rates of PERT prescription (21%) in patients with metastatic pancreatic cancer, in which the vast majority of patients had tumors of the pancreatic head and were likely to have had a blocked pancreatic duct [29].

It is unclear whether treatment of PEI with PERT improves the survival of patients with either resectable or unresectable pancreatic cancer. Therefore, the aim of this analysis was to evaluate the impact of PEI diagnosis and treatment on the survival of patients with unresectable pancreatic cancer.

## Methods

### Study design

This retrospective analysis was conducted at the University Hospital of Santiago de Compostela, Spain, on a database of patients with unresectable, pathologically confirmed, pancreatic adenocarcinoma.

### Study participants

Patients diagnosed with unresectable pancreatic cancer (locally advanced or metastatic disease) between January 2011 and October 2016, as evaluated by both computed tomography (CT) scan and endoscopic ultrasound (EUS), were eligible for inclusion in this analysis [30]. Confirmation of pancreatic adenocarcinoma by cytology or histology after EUS-guided fine needle aspiration (FNA) or fine needle biopsy (FNB) was required for final inclusion. EUS and EUS-guided FNA or FNB was performed in all patients by two experienced endosonographers using Pentax linear echoendoscopes and Hitachi Preirus (2011–2012) or Ascendus (2012–2016) equipment. All FNA and FNB samples were evaluated by a single highly experienced pathologist. Patients who survived less than 30 days after diagnosis, in whom no therapy could be started, and those who underwent neoadjuvant chemotherapy for downstaging, were excluded from the analysis.

#### Treatment

All patients were evaluated by medical oncologists for palliative chemotherapy and best palliative care, and the most appropriate tumor therapy according to age, performance status, liver and renal function, and general well-being was proposed to the patients. If obstructive jaundice was present, endoscopic biliary drainage was performed using a plastic or metal stent. In addition, the best supportive care for pain and symptoms, including nutritional supplements, was applied if needed.

Group 1 consisted of patients diagnosed with pancreatic cancer in the Department of Medical Oncology, General Surgery, or any department other than Gastroenterology, and who were treated according to well-accepted oncologic protocols [31]. Group 2 consisted of patients diagnosed with pancreatic cancer in the Department of Gastroenterology, and who were additionally followed-up at the Pancreas Unit of that Department and evaluated for symptoms of PEI, including significant weight loss (> 10% of body weight over less than 6 months before diagnosis), diarrhea and other maldigestion-related symptoms, and nutritional status. PERT was prescribed in patients in Group 2 if significant weight loss and/or other symptoms of PEI were present. Therefore, the management of patients was basically the same in the two groups except for evaluation of PEI and administration of PERT if needed in patients of Group 2.

#### Pancreatic exocrine insufficiency

As already mentioned, patients in Group 2 with symptoms suggestive of PEI were treated with PERT (Creon®, Abbott Laboratories GmbH, Germany), at an initial dose of 50,000 Eur.Ph.U with each main meal and 25,000 Eur.Ph.U. with snacks. This dose was individually titrated according to the evaluation of body weight, symptoms, and nutritional status. Omeprazole 20 mg was orally administered twice daily, before breakfast and dinner, in all patients with PEI. In addition, prokinetic drugs were prescribed three-times daily if dyspeptic symptoms (nausea, postprandial fullness, early satiety) were present.

#### Assessments

Demographics and clinical data including tumor stage, location and size, serum CA 19–9 levels, and weight loss (less or more than 10% of bodyweight) were recorded at diagnosis. Administration of chemotherapy and PERT were also recorded. Clinical and analytical follow-up of patients was performed according to medical needs. Patients in Group 2 were additionally assessed at the Department of Gastroenterology for: body weight; gastrointestinal symptoms, including those of PEI; nutritional status every 2–3 months. PERT was titrated if needed.

The survival of patients who received standard oncologic therapy alone (Group 1) was compared with that of patients who received the same therapy in addition to being evaluated and treated for PEI with PERT, if needed (Group 2). From this, the impact of PERT on survival was evaluated. All clinical information for each patient, including the data obtained for the study protocol, is accessible via a central electronic medical record, which is available to all the hospitals and health centers around the north-west region of Spain. All adverse events, newly diagnosed comorbidities, and deaths were prospectively recorded in this central electronic medical record.

### Statistical analyses

Descriptive data are shown as absolute numbers and percentages, median and range, and mean ± standard deviation (SD) as appropriate. Survival is shown as median and 95% confidence interval (CI). Baseline characteristics that could influence survival rates, such as age, gender, tumor stage, tumor location and size, serum CA 19–9 levels (dichotomized as > 1000 U/mL or < 1000 U/mL at diagnosis, or after biliary drainage in patients with obstructive jaundice), palliative chemotherapy and PERT (both dichotomized as “yes” or “no”), were studied as independent variables using a Cox proportional hazard regression model and expressed as hazard ratios (HR) (95% CI). Multivariate analysis was conducted using the forward-stepwise method, with *P* < 0.1 as a cut-off to enter into the model. Survival of different subgroups of patients was compared by the log-rank test and it was plotted as Kaplan–Meier curves using log-rank Mantel–Cox tests.

## Results

### Demographics

A total of 191 patients diagnosed with unresectable, pathologically-confirmed, pancreatic adenocarcinoma was identified during the study period. Thirty-one patients died within 30 days of diagnosis before any therapy could be started; thus, 160 patients were included in the final analysis. Mean age was 70.5 years (range 28–100); 92 patients were male (57.5%) and 68 were female (42.5%). Tumor staging revealed that 34 patients (21.2%) had locally advanced tumor and 126 (78.7%) had metastatic disease. The tumor was more frequently located in the head of the pancreas (58.1% of the cases, compared with 33.7% in the body and 8.1% in the tail of the pancreas). Tumor size was 4.2 ± 1.5 cm. Serum CA 19–9 levels at diagnosis and after biliary drainage were > 1000 U/mL in 73 patients (45.6%). Ninety-five patients (59.4%) had lost more than 10% of bodyweight within 1–6 months before diagnosis.

Of the 160 patients included, 86 (53.75%) were in Group 1 and 74 (46.25%) were in Group 2. Age, gender, tumor size, location and stage, weight loss, and serum CA 19–9 levels were similar in both groups (Table 1).

**Table 1:** Demographics and clinical data of patients with unresectable pancreatic cancer according to the study group

	Group 1	Group 2	*P* value
n (%)	86 (53.75%)	74 (46.25%)	
Age at diagnosis, median (range), years	71.5 (43–100)	69.5 (28–90)	0.470^c^
Gender (M/F)	52/34	40/34	0.427
Tumor Stage (locally advanced/metastatic)	16/70	18/56	0.440
Tumor location (head/body/tail)	46/33/7	47/21/6	0.396
Tumor size, mean ± SD (cm)	4.5 ± 1.5	4.1 ± 1.5	0.828^d^
Patients with CA 19–9 > 1000 U/mL at diagnosis^a^	40 (55.5%)	33 (47.8%)	0.401
Weight loss > 10% BW at diagnosis, n (%)	47 (54.7%)	48 (64.9%)	0.220
Patients receiving palliative chemotherapy, n (%)	40 (46.5%)	53 (71.6%)	0.001
Number of chemotherapy cycles, median (range)^b^	3 (0.3–20)	4 (1–40)	0.004^c^
Survival, median (range), days	95.0 (33–768)	189 (31–997)	< 0.001^c^
Patients receiving PERT, n (%)	0 (0%)	49 (66.2%)	< 0.001

^a^After biliary drainage in patients with obstructive jaundice (data available in 88.1% of patients)

^b^Patients receiving chemotherapy (*n* = 93)

^c^Mann–Whitney U test

^d^Student-t test

### Treatment

Ninety-three patients (58.1%) were clinically judged to be suitable for, and accepted, palliative chemotherapy; 46.5% in Group 1 and 71.6% in Group 2 (*P* < 0.001). Of the 53 patients in Group 2 receiving chemotherapy, 13 were initially considered unsuitable, but were later suitable and treated with chemotherapy after receiving other supportive therapies, including PERT. Chemotherapy regimens were selected following standard guidelines [31] according to age and performance status of the patients, and included gemcitabine monotherapy, gemcitabine-Nab-paclitaxel, folfirinox, and folfox. Sixty-seven patients were unfit for chemotherapy and only received best palliative clinical care.

Pancreatic enzyme replacement therapy was prescribed for 49 patients in Group 2 (30.6% of the total patient population; 66.2% of the patients in Group 2), at a median daily dose of 325,000 Ph.Eur. (75,000 Ph.U. with main meals, 50,000 Ph.U. with snacks), ranging from 200.000 to 400,000 Ph.Eur. daily. Forty of the 49 patients receiving PERT (81.6%) had reported significant weight loss (> 10% bodyweight within 6 months) at diagnosis.

### Survival

**Fig. 1:**
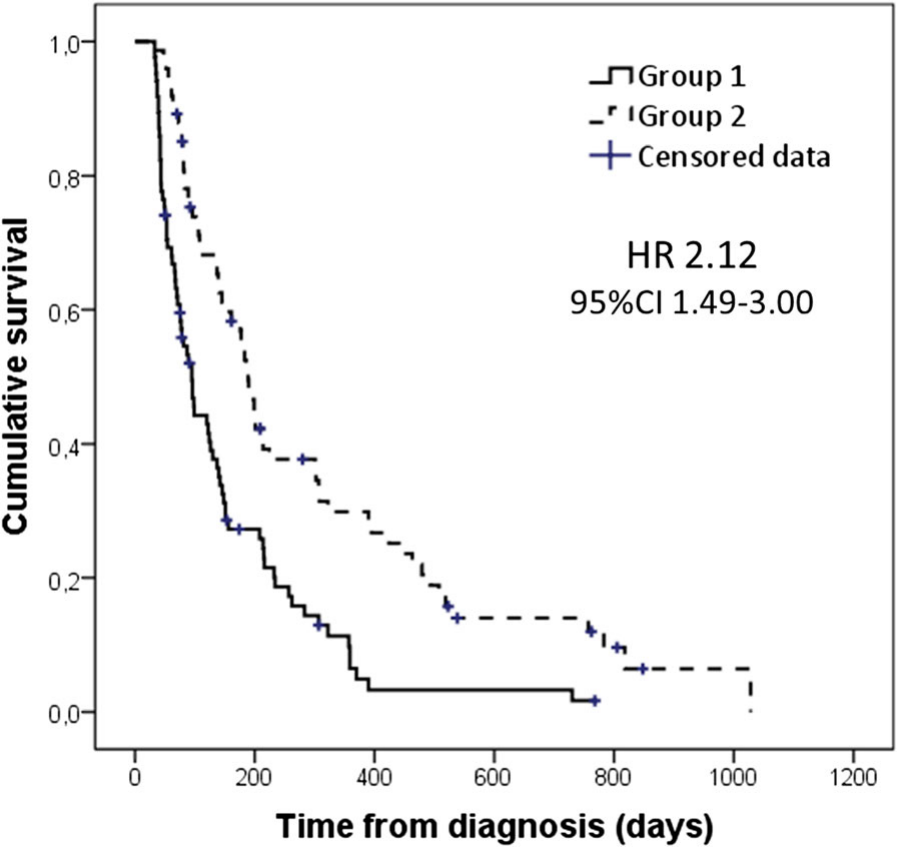
Kaplan–Meier survival curves of patients with unresectable pancreatic cancer in Group 1 (standard oncologic therapy, *n* = 86) and Group 2 (evaluation of symptoms of pancreatic exocrine insufficiency [PEI] and the need for pancreatic enzyme replacement therapy [PERT] in addition to standard oncologic therapy, *n* = 74). The hazard ratio (HR) and 95% confidence interval (CI) associated with Group 2 are shown. Log-rank test (Mantel–Cox) *P* < 0.001

The overall mortality rate was 86.9% (139/160 patients) at the end of the study period. Median survival was 139 days (95% CI 110–168 days), and was longer in patients with locally advanced tumors (257 days, 95% CI 125–389 days) than in those with metastatic disease (126 days, 95% CI 95–157 days) (*p* = 0.007). Survival in Group 2 (189 days, 95% CI 167.0–211.0 days), in which presence of PEI and need of PERT was evaluated additionally to standard oncologic therapy, was significantly longer than in Group 1 (95.0 days, 95% CI 75.4–114.6 days), in which only standard oncologic therapy was given (HR 2.12, 95% CI 1.49–3.00; *P* < 0.001) (Fig. 1).

**Fig. 2:**
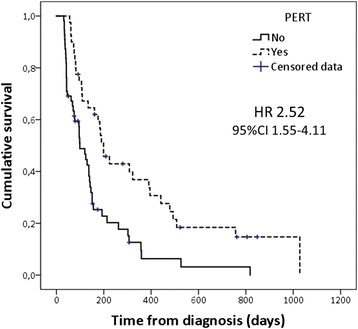
Kaplan–Meier survival curves of patients with unresectable pancreatic cancer and a significant weight loss (> 10% bodyweight within 6 months) at diagnosis (*n* = 95) according to administration of pancreatic enzyme replacement therapy (PERT). The hazard ratio (HR) and 95% confidence interval (CI) associated with PERT are shown. Log-rank test (Mantel–Cox) *P* < 0.001

In the univariate and multivariate analysis, chemotherapy and PERT were significantly and independently associated with longer survival in a model that included age and tumor stage (Table 2). In Group 2, the survival of patients treated with PERT due to symptoms suggestive of PEI was similar to that of those who did not receive PERT due to absence of PEI (HR 1.12, 95% CI 0.67–1.89). In contrast, if all patients from Groups 1 and 2 with significant weight loss at diagnosis (> 10% bodyweight within 6 months) were considered (*n* = 95), the survival of those who received PERT (*n* = 40) was significantly longer than that of those who did not (median survival 199 days [95% CI 152–246] versus 99 days [95% CI 68–130 days]; HR 2.52, 95% CI 1.55–4.11, *P* < 0.001) (Fig. 2).

**Table 2:** Analysis of factors associated with survival among patients with unresectable pancreatic cancer (*n* = 160) (Cox proportional hazard regression analysis)

	Univariate analysis	*P* value	Multivariate analysis	*P* value
	HR (95% CI)		HR (95% CI)	
Age at diagnosis	1.03 (1.01–1.05)	< 0.001	1.02 (1.00–1.03)	0.072
Gender				
Female	1.00	0.517		
Male	0.89 (0.63–1.26)			
Tumor stage				
Locally advanced tumor	1.00	0.008	1.00	0.001
Metastatic disease	0.56 (0.37–0.86)		0.47 (0.30–0.72)	
Tumor location				
Head	1.00	0.202		
Body/tail	1.25 (0.89–1.76)			
Tumor size (cm)	0.97 (0.84–1.13)	0.733		
CA 19–9 levels at diagnosis				
< 1000 U/mL	1	0.143		
> 1000 U/mL	0.76 (0.53–1.10)			
Weight loss at diagnosis				
< 10% BW	1	0.420		
> 10% BW	0.87 (0.62–1.22)			
Chemotherapy	4.68 (3.18–6.89)	< 0.001	3.69 (2.33–5.85)	< 0.001
PERT	1.80 (1.24–2.62)	0.002	1.81 (1.23–2.66)	0.002

*BW* body weight, *CI* confidence interval, *HR* hazard ratio, *PERT* pancreatic enzyme replacement therapy

## Discussion

This retrospective analysis conducted in patients with unresectable pancreatic cancer (locally advanced or metastatic disease) showed that the evaluation and treatment of PEI, in addition to standard chemotherapy and best supportive care, was associated with significantly prolonged survival compared with standard chemotherapy alone (189 versus 95 days, respectively, *P* < 0.001). This is especially true for patients with significant weight loss of more than 10% of their bodyweight at diagnosis. Palliative chemotherapy and PERT were independently associated with longer survival.

The incidence of pancreatic cancer is projected to increase to become the second most common cancer by 2030 [2], yet the overall survival rate remains low [1]. Around 80% of patients present with unresectable, advanced disease, which has a median survival of just 6–12 months [4, 13]. Therefore, for this patient population, in which curative surgery is not possible, palliative and supportive care is key to prolonging survival and improving quality of life.

Cachexia can directly reduce the quality of life of patients with cancer, and pancreatic cancer often causes a high prevalence of severe cachexia [32]. Cachexia has previously been shown to be associated with short survival and poor quality of life in patients with pancreatic cancer [5, 19]. Furthermore, tolerance to chemotherapy is reduced in patients with cachexia and malnutrition [33].

In patients with pancreatic cancer, weight loss is obviously multifactorial, but PEI plays a major role [5, 34]. PEI can develop after surgery or may be caused by the tumor obstructing the pancreatic duct [22, 24]. PEI, the presence of metastases, anemia, and hypoalbuminemia are all independent predictors of survival in patients with advanced pancreatic cancer [26]. Furthermore, PEI has been shown to negatively impact the quality of life of patients with pancreatic cancer, mainly due to its associated difficulties in managing diet and gastrointestinal symptoms [35]. The positive impact of PERT on survival reported in the present study in patients with unresectable pancreatic cancer and associated significant weight loss supports the relevant role of PEI in these patients. Conversely, PERT may have no benefit in patients with no significant weight loss.

Pancreatic enzyme replacement therapy, the standard treatment for PEI [23], has been shown to improve weight gain in patients with advanced pancreatic cancer by improving digestion and nutrient absorption [16]. This analysis shows that these improvements are reflected in improved survival rates in patients with unresectable pancreatic cancer who received PERT for PEI, together with standard oncologic therapy, compared with those who received oncologic therapy alone. Interestingly, the rate of survival of patients with PEI can be increased to the level of patients without PEI by giving PERT; this suggests that the enzyme doses used in this study (starting at 200000 Ph.Eur. per day, followed by titration up to a median dose of 325,000 Ph.U. per day according to body weight, symptoms, and nutritional status during follow-up) are effective in treating PEI in these patients. It should be underlined that PERT is well tolerated with mild and infrequent side effects [36], which is especially important for patients with pancreatic cancer. In this analysis, no patient discontinued PERT due to side effects or intolerance.

In the present study, omeprazole was consistently administered to patients with PEI to improve the efficacy of PERT due to the resultant inhibition of gastric acid secretion. It is well known that PEI is not only the consequence of reduced pancreatic secretion of enzymes, but also of reduced bicarbonate secretion [37]. Pancreatic enzymes in enteric-coated preparations require a pH higher than 5.5 for them to be released [38]. Intestinal pH is frequently acidic in patients with PEI, thus avoiding the release of exogenous enzymes within the proximal gut [39]. Addition of a proton pump inhibitor to PERT has been shown to be effective in improving fat digestion in patients with PEI [40]. This is especially important in patients with unresectable pancreatic cancer in whom pancreatic secretion is markedly reduced due to the obstruction of the main pancreatic duct.

Palliative chemotherapy was strongly associated with a longer survival in this study. Nevertheless, only patients who were clinically judged to be suitable for chemotherapy were administered this treatment, whereas those who were unsuitable with a poor performance status were not administered chemotherapy. Thus, there is a very relevant bias in the selection of patients for chemotherapy, and it is not appropriate to use this study to evaluate the efficacy of this form of therapy. Interestingly, the proportion of patients in this study that were suitable for chemotherapy and the number of therapy cycles that these patients tolerated increased with PERT, thus suggesting that PERT may increase tolerance to chemotherapy.

The retrospective study design is the main limitation of the present study; however, the analysis was limited to hard objective variables that are well-recorded in clinical practice. In addition, this study used data from a single central electronic medical record of mandatory use for all the physicians in hospitals and health centers in the north-west region of Spain. This electronic medical record includes information on any medical assistance, as well as any laboratory tests, imaging, and any other examination performed. The use of this central electronic medical record markedly reduces the possibility of missed data. The limited number of patients included is also a limitation in this study, and is mainly due to the single-center study design and the need for pathological confirmation of pancreatic adenocarcinoma for inclusion. The single-center design limits the variability in patients’ care and therapy, and the requirement of pathological confirmation for inclusion avoids biases related to the inclusion of other malignant lesions. These two features could be therefore considered strengths of the study.

Prospective clinical trials are needed to confirm the impact of PERT in patients with unresectable pancreatic cancer, not only on survival, but also on their quality of life and tolerance to chemotherapy. A recent Phase II trial found that the mean percentage change in body weight of patients with unresectable pancreatic cancer did not differ significantly between those randomized to receive PERT or placebo for 8 weeks [41]; however, this study had several limitations. The limitations included: a small sample size; short follow-up period; low enzyme dose (50,000 Ph.Eur. with main meal and 25,000 Ph.Eur. with snacks); tablets were used and not minimicrospheres; and finally, FE-1 levels were above the normal threshold of 200 μg/g stool in 44.1% of patients in the PERT group and 21.2% of those in the placebo group [41]. Larger, well-designed, randomized trials are needed to identify patients who may benefit from PERT.

It is important to note that PERT is generally accepted for the treatment of PEI of any etiology, and until new data are available, it should be considered as part of the therapeutic armamentarium in patients with pancreatic cancer. This is important not only in clinical practice, but also in clinical trials for new chemotherapy agents. The possibility of PEI and its treatment with PERT in patients with pancreatic cancer has been consistently ignored in previous clinical trials of chemotherapy agents. The present study suggests that PERT increases tolerance to chemotherapy, and opens the question of whether the efficacy of standard chemotherapeutic agents could be improved by the inclusion of the evaluation and treatment of PEI with PERT as part of the best standard of care in these patients.

## Conclusions

This study showed that evaluation of PEI and its treatment with PERT can prolong survival in patients with unresectable pancreatic cancer. PEI and its treatment should be taken into consideration as part of the best standard of care in these patients, and when assessing future oncology treatment to increase patient survival and quality of life in this population.

## Abbreviations

BW: Body weight
CI: Confidence interval
CT: Computed tomography
EUS: Endoscopic ultrasound
FE-1: Fecal elastase-1
FNA: Fine needle aspiration
FNB: Fine needle biopsy
HR: Hazard ratio
PEI: Pancreatic exocrine insufficiency
PERT: Pancreatic enzyme replacement therapy
SD: Standard deviation
